# 

**Published:** 1880-11

**Authors:** 


### CONTENTS:

ART. I.—Some Points in the Natural History of Caries of the Teeth, and the Value of Fillings for its arrest. By G. V. Black, D. D. S. .......... 289
ART. II.—Progress of Dental Surgery in the United States. By Dr J. B. Patrick .......... 308
ART. III.—A Method of Capping Exposed Pulps, with Cases in Practice. By Dr. M. S. Jobson .......... 315
ART. IV.—Vulcanizer Explosions .......... 320
ART. V.—Prophylaxis, or Prevention of Dental Decay. By C. C. Patrick, D. D. S. .......... 325

### EDITORIAL, ETC.

Maryland and District of Columbia Dental Association .......... 329
To Our Young Men—A Caution .......... 330
Socket Instruments .......... 330

### MONTHLY SUMMARY.

Unerupted Teeth in the Roof of the Mouth .......... 331
Another Discovery .......... 332
Behaviour of Metals when Heated in Vacuo .......... 333
Disturbances of Vision by Unsound Meat .......... 333
Spence's Metal .......... 334
The Value of Prolonged Artificial Respiration .......... 335
Glass Wicks for Lamps .......... 335
Anæsthetics in 1651 .......... 335
Motion in Teeth .......... 336
Nitro-Glycerine .......... 336

